# Complete Genome Sequences of Dengue Virus Type 2 Epidemic Strains from Reunion Island and the Seychelles

**DOI:** 10.1128/MRA.01443-19

**Published:** 2020-01-23

**Authors:** Hervé Pascalis, Leon Biscornet, Céline Toty, Sarah Hafsia, Marjolaine Roche, Philippe Desprès, Célestine Atyame Nten, Jastin Bibi, Meggy Louange, Jude Gedeon, Patrick Mavingui

**Affiliations:** a UMR Processus Infectieux en Milieu Insulaire Tropical, Université de La Réunion, CNRS 9192, INSERM U1187, IRD 249, Plateforme de Recherche CYROI, Sainte Clotilde La Réunion, France; b Infectious Disease Surveillance Unit, Seychelles Public Health Laboratory, Public Health Authority, Ministry of Health, Victoria, Seychelles; c Disease Surveillance and Response Unit, Epidemiology and Statistics Section, Public Health Authority, Ministry of Health, Victoria, Seychelles; KU Leuven

## Abstract

Dengue virus has recently reemerged in the southern Indian Ocean islands, causing outbreaks in Reunion Island and the Seychelles. In the present study, we determined the complete genome sequences of closely related clinical isolates of dengue virus type 2 circulating in the Seychelles in 2016 and Reunion Island in 2018.

## ANNOUNCEMENT

Like many tropical regions in which competent mosquito vectors are present, the islands of the southwestern Indian Ocean have experienced a renewal of human pathogenic arbovirus emergence (1, 2). Indeed, dengue virus type 2 (DENV-2), belonging to the *Flavivirus* genus (*Flaviviridae* family), has reemerged, causing outbreaks in Reunion Island and the Seychelles. Between December 2017 and April 2019, 49,000 DENV-2-infected cases with 428 hospitalizations were recorded in Reunion Island (3, 4). In the Seychelles, 1,967 suspected cases with 215 hospitalizations were reported between January 2015 and December 2016 (5).

Here, we report the whole-genome sequencing of two DENV-2 strains, namely, RUJul, isolated from Saint-Gilles, Reunion Island, in 2018, and RU16417, isolated from Victoria, Seychelles, in 2016. Sera were collected from patients with dengue symptoms (fever, myalgia, headache, asthenia, and thrombocytopenia) and without recent travel history. RNA was extracted using the QIAamp kit (Qiagen). cDNA was synthesized using ProtoScript II with random primers (New England) and was purified using the QIAquick kit (Qiagen). Libraries were prepared with the Nextera XT kit (Illumina) and subjected to paired-end sequencing (2 × 250 bp) on an Illumina HiSeq 4000 system (Genoscreen). The total read counts were approximately 1,034,620 (RUJul) and 622,542 (RU16417). The read quality was assessed by FastQC (6). Adapters and leading and trailing low-quality bases (Q scores of <30) or N bases were removed using Trimmomatic software (7). Trimmed reads, ranging from 64 to 250 bases, were assembled by mapping to a reference Thai DENV-2 genome (GenBank accession number NC_001474) using Bowtie2 v2.1.0 (8). The numbers of reads that mapped to the reference genome were 498,356 (RUJul) and 264,288 (RU16417), giving coverage of 7× and 15×, respectively, with similar GC contents of 45%. Untranslated regions (UTRs) were further amplified by rapid amplification of cDNA ends (RACE) PCR using the SMARTer RACE 5ʹ/3ʹ kit (TaKaRa) with RNA extracted from culture supernatants of Vero E6 cytopathic cells resulting from infection with viremic patient sera (passage 1), and they were then Sanger sequenced (Genoscreen).

**FIG 1:**
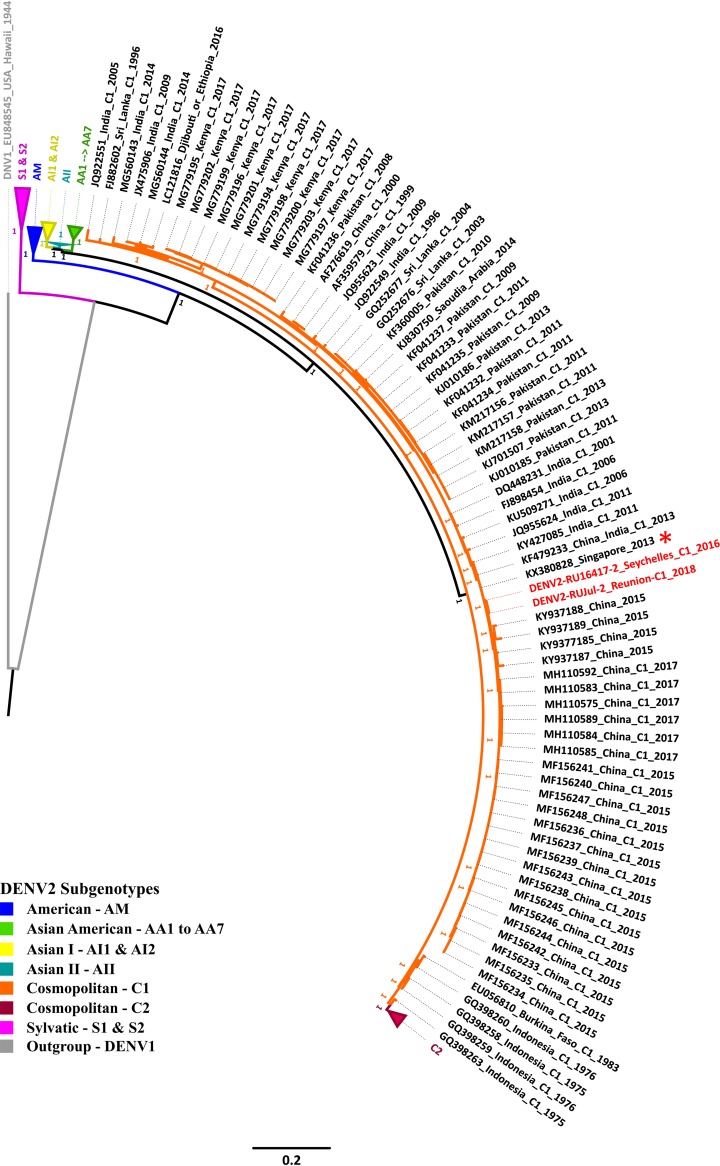
Phylogenetic tree analysis of DENV-2 strains. PhyML v3.0 software (12) was used to produce a maximum likelihood tree with the best-fit nucleotide substitution model (GTR+G+I), with complete genome sequences (*n* = 230) of selected reference strains. Support values were calculated using the Bayes method implemented in PhyML. The clades of the other subgenotypes have been collapsed for clarity. A DENV-1 sequence was included as an outgroup. The scale bar indicates nucleotide substitutions per site. Strains in the tree are reported with the following information: GenBank accession number, country, subgenotype, and collection date. The two strains reported in this study are shown in red, and the closest sequence (GenBank accession number KX380828) is represented by a red asterisk. The phylogenetic tree was edited with FigTree v1.4.4 software (http://tree.bio.ed.ac.uk/software/figtree).

UTRs of 93 nucleotides (5ʹ UTR) and 452 nucleotides (3ʹ UTR) were manually added, and the resulting genomic sequences of about 10,724 nucleotides were aligned with a DENV reference set and annotated using Geneious (9). BLASTN analysis with multiple alignment revealed that the two genomes shared 99.8% identity and exhibited 93% identity with the genomic sequence of a DENV-2 strain from Thailand (GenBank accession number KX380828). Phylogenetic analysis of the two genomes with full sequences available for representative DENV strains (10) positioned the RUJul and RU16417 strains within DENV-2 Cosmopolitan genotype lineage I (Fig. 1), confirming previous results (11). Genome annotation identified the expected polyprotein of about 3,754 amino acids, including 3 structural proteins (C, prM/M, and E) and 7 nonstructural proteins (NS1, NS2A, NS2B, NS3, NS4A, NS4B, and NS5) flanked by the UTRs described. Despite dengue virus outbreaks in the southwestern Indian Ocean, few complete genomes are currently available. The DENV-2 genomes provided here will allow better assessment of the evolutionary epidemiological history, considering the specific regional contexts regarding viral introduction, circulation, and stages of endemicity.

### Data availability.

The assembled genomes were deposited in GenBank under accession numbers MN272404 (RUJul) and MN272405 (RU16417). Raw data are available in the NCBI SRA under BioProject accession number PRJNA575805.
